# Long Blood Residence and Large Tumor Uptake of Ruthenium Sulfide Nanoclusters for Highly Efficient Cancer Photothermal Therapy

**DOI:** 10.1038/srep41571

**Published:** 2017-01-31

**Authors:** Zhuoxuan Lu, Feng-ying Huang, Rong Cao, Liming Zhang, Guang-hong Tan, Nongyue He, Jie Huang, Guizhen Wang, Zhijun Zhang

**Affiliations:** 1 Key Laboratory of Tropical Diseases and Translational Medicine of the Ministry of Education, Hainan Medical College, Haikou 571101, China; 2 Department of Chemical Engineering, Monash University, Wellington Rd., Clayton, Vic 3800, Australia; 3 Hunan Key Laboratory of Green Chemistry and Application of Biological Nanotechnology, Hunan University of Technology, Zhuzhou 412008, China; 4 Key Laboratory of Nano-Bio Interface, Division of Nanobiomedicine, Suzhou Institute of Nano-tech and Nano-bionics, Chinese Academy of Sciences, Suzhou 215123, China; 5 College of Materials and Chemical Engineering, Hainan University, Haikou 570228, China

## Abstract

Transition metal sulfide (TMS) holds great potential in cancer photothermal therapy (PTT) because of the high absorbance in the near-infrared (NIR) region. The short blood circulation time and limited tumor accumulation of TMS-based photothermal agents, however, limit their applications. Herein, we design a novel TMS-based PTT agent, ruthenium sulfide-based nanoclusters (NCs), to overcome the current limitations. We firstly develop a simple method to prepare oleic acid coated ruthenium sulfide nanodots (OA-RuS_1.7_ NDs) and assemble them into water-soluble NCs *via* sequentially coating with denatured bovine serum albumin (dBSA) and poly(ethylene glycol) (PEG). The obtained PEG-dBSA-RuS_1.7_ NCs possess excellent photothermal conversion ability. More significantly, they exhibit enhanced blood circulation time and tumor-targeting efficiency *in vivo* compared with other TMS-based PTT nanoagents, which may be attributed to their appropriate hydrodynamic diameter (~70 nm) and an ideal charge (~0 mV). These characteristics help the PEG-dBSA-RuS_1.7_ NCs to escape the removal by the reticuloendothelial system (RES) and kidney. All these advantages enable the PEG-dBSA-RuS_1.7_ NCs to selectively concentrate in tumor sites and effectively ablate the cancer cells upon NIR irradiation.

Recently, many novel therapeutic strategies have been explored for tumor treatment since conventional therapies have a lot of shortcomings^1^,^2^,^3^,^4^,^5^,^6^. Among them, photothermal therapy (PTT) shows great promise because of its improved therapeutic efficacy, spatiotemporal controllability, low systemic toxicity, and limited side effects^7^,^8^,^9^,^10^. In PTT, photothermal agents are targeting delivered to the tumor area and then the tumor is laser irradiated, which leads to a temperature rise in tumor area and destroys cancer cells. Considering that near-infrared (NIR, λ = 700–1000 nm) laser has high depth of penetration into bio-tissues, numerous photothermal agents that can efficiently convert NIR light into heat for cancer treatment. have been proposed^11^,^12^,^13^,^14^. Some photothermal agents with high NIR absorption for PTT have been studied in detail, such as gold nanorods^15^, gold nanoshells^16^, carbon-based nanomaterials^17^,^18^ and organic compounds^19^.

In the past decades, metal sulfide nanomaterials, especially transition metal sulfide (TMS) nanomaterials, have been widely studied for many potential applications by virtue of excellent electronic, optical, and mechanical properties^7^,^20^,^21^,^22^. Currently, the applications of TMS has been expanded to PTT due to the strong NIR absorbance. For instance, MoS_2_ nanosheet-based multifunctional agents were developed for combined photothermal therapy and chemotherapy of cancers^20^,^23^; Bi_2_S_3_ nanorods were used as a novel nanomedicine for imaging-guided tumor PTT^24^; WS_2_ nanoflakes were explored for cancer treatment^25^. These newly emerging photothermal agents are promising in cancer theranostics, but some inherent limitations remain central concerns for clinical applications. More specifically, many of previously reported TMS-based nanoagents for PTT were prepared by exfoliation of their bulk counterparts, which often require complex fabrication process^23^. Moreover, the short blood circulation time and limited tumor accumulation largely restrict the *in vivo* applications of TMS-based nanomedicines. For example, it has been reported that the accumulated amount of Tween-Bi_2_S_3_ nanorods in liver and spleen is more than 10 times higher than that in tumor possibly by the clearance *via* the reticuloendothelial system (RES)^24^. Thus, it is essential to develop new TMS-based photothermal agents with long blood residence and high tumor uptake by a facile preparation route.

As a member of TMS, ruthenium sulfide with a similar band gap as MoS_2_ (~1.8 eV) may make itself suitable to be a NIR-absorber^26^,^27^, but until now it has not yet been used in PTT. Therefore, we wonder if ruthenium sulfide can be applied as an ideal PTT agent with prolonged blood circulation and enhanced uptake by in tumors. Following this thought, here ruthenium sulfide nanodots (NDs) with a diameter of ~1.5 nm was firstly prepared *via* a simple method, and the molar ratio of Ru and S was determined to be 1:1.7, accordingly referred as RuS_1.7_. Since nanoparticles with hydrodynamic diameter between 20–100 nm, especially in the size range of 60–80 nm, can efficiently avoid being removed by the RES and excreted by kidney^28^,^29^, we assembled the RuS_1.7_ NDs to nanoclusters (NCs) by sequential coating with denatured bovine serum albumin (dBSA) and poly(ethylene glycol) (PEG) to keep the size within the expected range. The obtained PEG-dBSA-RuS_1.7_ NCs show strong absorption in NIR region, excellent photothermal conversion ability, good dispersibility and stability in physiological solution, as well as negligible toxicity *in vitro* and *in vivo*. Most importantly, the as-prepared PEG-dBSA-RuS_1.7_ NCs display long blood circulation time and high tumor accumulation level *in vivo*. Thus, our report provides a powerful strategy for developing tumor-targeting TMS-based PTT agents.

## Results and Discussion

### Synthesis and characterization of RuS_1.7_ NDs and PEG-dBSA-RuS_1.7_ NCs

**Figure 1:**
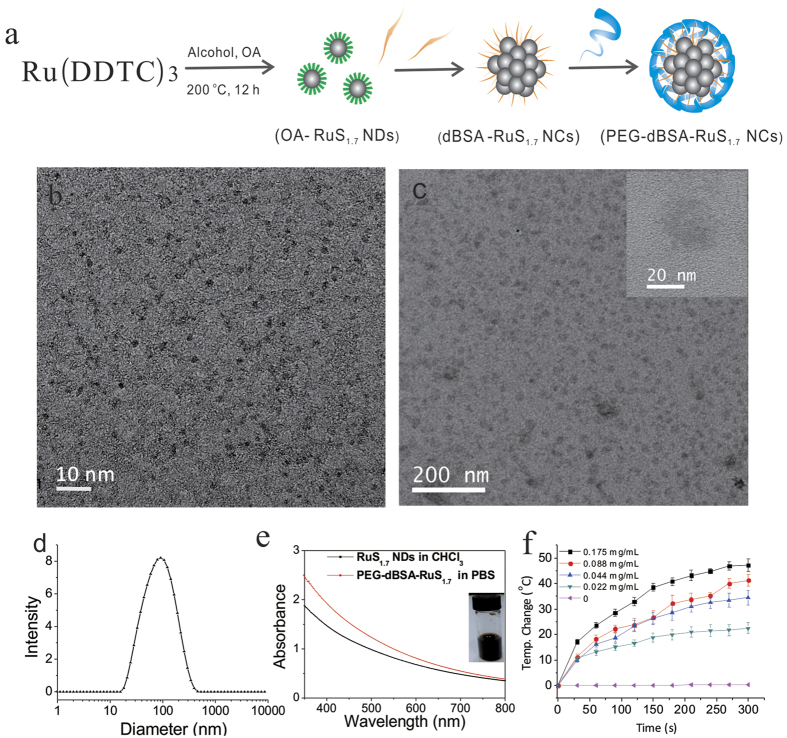
Preparation and characterization of RuS_1.7_ NDs and PEG-dBSA-RuS_1.7_ NCs. (**a**) Schematic illustration of the preparation and modification of RuS_1.7_ NDs. (**b**) TEM image of OA-RuS_1.7_ NDs. (**c**) TEM image of PEG-dBSA-RuS_1.7_ NCs. (**d**) DLS-measured the diameter of as-prepared PEG-dBSA-RuS_1.7_ NCs in water. (**e**) Vis-NIR spectrums of OA-RuS_1.7_ NDs and PEG-dBSA-RuS_1.7_ NCs. Inset: Photograph of the PEG-dBSA-RuS_1.7_ NC solution in PBS. (**f**) Photothermal heating curves of PEG-dBSA-RuS_1.7_ NCs with 0.5 mL of solution volume and 1.4 W cm^−2^ of power density.

The preparation of RuS_1.7_ NDs and the subsequent coating with dBSA and PEG is illustrated in Fig. 1a. We prepared RuS_1.7_ NDs *via* a facile solvothermal method by decomposing the diethyl dithiocarbamate ruthenium (Ru(DDTC)_3_) dissolved in the mixture of oleic acid (OA) and ethyl alcohol (V:V = 2:1) in a Teflon-lined autoclave. X-ray fluorescence (XRF) spectroscopy, X-ray diffraction (XRD), and X-ray photoelectron spectroscopy (XPS) were conducted to confirm the chemical composition of the as-synthesized RuS_1.7_ NDs. The XRF spectrum demonstrates the characteristic fluorescence peaks of Ru and S (Fig. S1). The molar ratio of Ru to S was calculated to be 1:1.7 (weight ratio of Ru to S is 65:35) based on the intensity of the fluorescence peaks of Ru and S. As can be seen in Fig. S2, different from that of polycrystalline ruthenium sulfide (Joint Committee on Powder Diffraction Standards, File No. 19–1107), the XRD pattern only shows broad humps, which indicates that the as-obtained RuS_1.7_ NDs are in an amorphous phase. To further investigate the composition and oxidation state of the RuS_1.7_ NDs, we performed XPS characterization. In the Ru 3d spectrum (Fig. S3), two peaks centered at 279.7 and 283.8 eV represent the 3d_5/2_ and 3d_3/2_ states, with the range of the binding energy for Ru^2+^. The sulfur 2p doublet with peaks located near 162.0 eV falls into the range of the binding energy for (S_2_)^30^,^31^. Our results are in good agreement with previous report of RuS_1.7_ by Jeevanandam *et al*.^30^.

For PTT applications, good dispersibility in physiological solutions is of great importance. Because the synthesized RuS_1.7_ NDs coated by OA are well dispersed in many organic solvents but not dissolved in aqueous solutions, we further functionalized them with dBSA and PEG. In this way, RuS_1.7_ NDs were aggregated to NCs. The formed NCs are water-soluble, and fourier-transform infrared (FTIR) spectroscopy was used to determine the coating of RuS_1.7_ NDs. The bands at 1625 cm^−1^ and 2988 cm^−1^ in the FTIR spectrum of OA-RuS_1.7_ NDs can be assigned to the characteristic vibration of carbon-carbon double bond of OA and the vibration of unsaturated carbon-hydrogen bond from OA, respectively (Fig. S4)^24^. These characteristic bands of OA disappeared after modification with dBSA followed by the conjugation with PEG, while an obvious new band at 1650 cm^−1^ ascribed to the vibration of carbonyl group from amide of dBSA was recorded (Fig. S4)^23^. Also, typical transmission electron microscope (TEM) and hydrodynamic size were employed to evaluate our PTT agent since a suitable size is beneficial for tumor-specific accumulation. As illustrated in Fig. 1b, the as-synthesized RuS_1.7_ NDs show a uniform size with ~1.5 nm in diameter. After sequentially modified by dBSA and PEG, the clusters were formed by the aggregation of the RuS_1.7_ NDs with the diameter ~23 nm (Figs 1c and S5). The formed NCs exhibited well stability in water and PBS, and do not precipitate within at least 6 months even in high concentration (2 mg mL^−1^) (Fig. S6). As shown in Fig. 1d, the hydrodynamic size of the PEG-dBSA-RuS_1.7_ NCs measured by dynamic light scattering (DLS) is 70 nm with a polydispersity index of 0.226. This size of nanoparticles is well suited for prolonging blood circulation time and improving tumor accumulation^28^,^29^. In addition, the zeta potential value of PEG-dBSA-RuS_1.7_ NCs (close to 0 mV) can effectively make themselves “stealthy” thereby evading the recognition by RES (Fig. S7)^28^.

**Figure 2:**
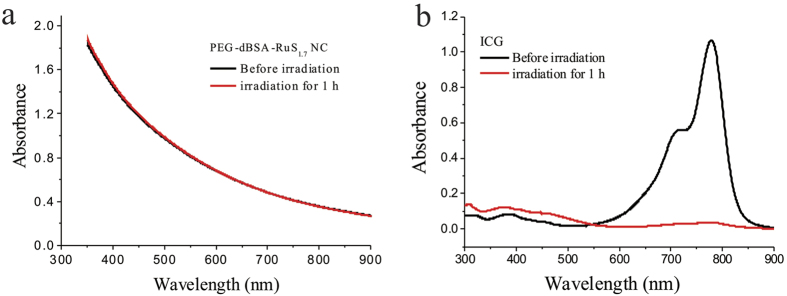
Vis-NIR spectrums of (**a**) PEG-dBSA-RuS_1.7_ NC solution and (**b**) ICG solution before and after irradiation using 808 nm laser with power density of 1.4 W cm^−2^ for 1 h.

Next, we studied NIR absorption and photothermal performance of PEG-dBSA-RuS_1.7_ NCs. As shown in Fig. 1e, Vis-NIR spectrums indicate that RuS_1.7_ NDs have a very broad absorption in the NIR region, which is not affected by the sequential modification with dBSA and PEG. The mass extinction coefficient of PEG-dBSA-RuS_1.7_ NCs at 800 nm was determined to be 9.4 L g^−1^ cm^−1^, much higher than that of graphene oxide used in cancer PTT (λ = 800 nm, 3.6 L g^−1^ cm^−1^)^32^. To survey the photothermal performance of our nanoclusters, the PEG-dBSA-RuS_1.7_ NCs solutions of different concentrations were exposed to an 808 nm continuous-wave laser at a power density of 1.4 W cm^−2^ (Fig. 1f). The photothermal heating curves recorded by an IR thermal camera show a strong concentration-dependent photothermal effect with the highest temperature increment up to 47.2 °C. In contrast, only a slight temperature rise (~3 °C) was observed for water. According to the reported method^8^,^24^,^33^, the photothermal conversion efficiency of PEG-dBSA-RuS_1.7_ NCs was calculated to be ~28.5% which was similar to that of Bi_2_S_3_ NRs (~28.1%)^24^. Besides, the study on photostability of PEG-dBSA-RuS_1.7_ NCs was carried out. The Vis-NIR spectrum of PEG-dBSA-RuS_1.7_ solution after the continuous irradiation for 1 h, shows no noticeable change, while the Vis-NIR absorption of indocyanine green (ICG) is largely decreased (Fig. 2a and b). The excellent photothermal effects and photothermal stability of PEG-dBSA-RuS_1.7_ NCs make themselves highly potential to be developed as a photothermal therapeutic agent.

### *In vitro* and *in vivo* toxicity of PEG-dBSA-RuS_1.7_ NCs

**Figure 3:**
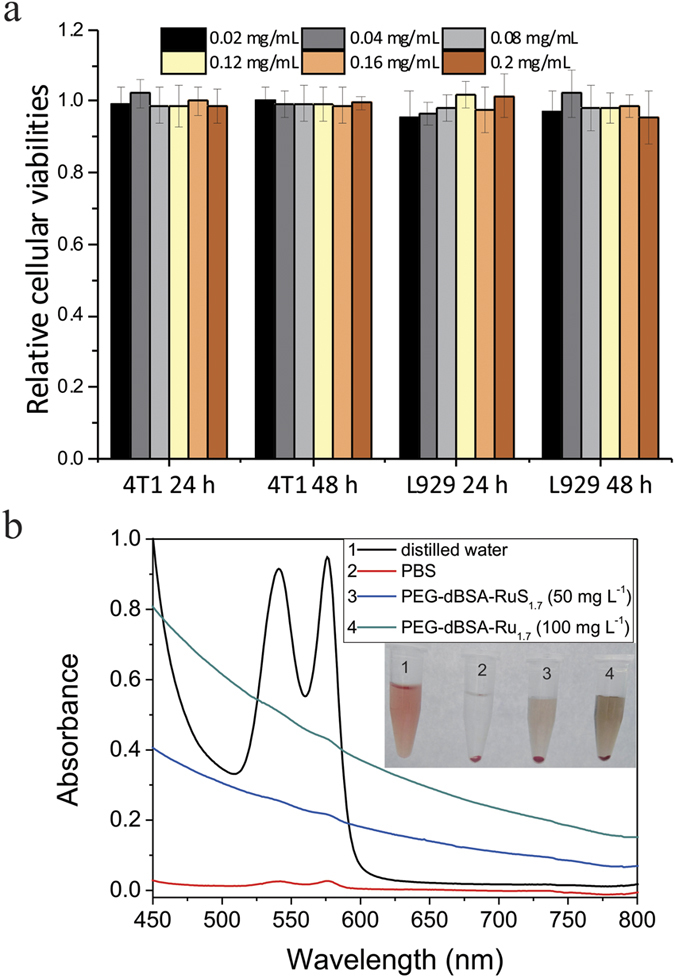
*In vitro* and *in vivo* toxicity of PEG-dBSA-RuS_1.7_ NCs. (**a**) Relative cellular viabilities of PEG-dBSA-RuS_1.7_ NC-treated cells. (**b**) Hemolytic analysis of PEG-dBSA-RuS_1.7_ NCs to red blood cells.

In order to use PEG-dBSA-RuS_1.7_ NCs in PTT, firstly the *in vitro* and *in vivo* toxicity of them should be evaluated. In our study, a standard WST-1 cell proliferation assay^34^,^35^ was conducted using the mouse mammary tumor cell line (4T1) and fibroblast cell line (L929). After the cells were incubated with various concentrations of PEG-dBSA-RuS_1.7_ NCs for 24 or 48 h, we found that the relative cellular viabilities did not obviously decrease for PEG-dBSA-RuS_1.7_ NC-treated cells even at the highest nanocluster concentration (200 μg mL^−1^) (Fig. 3a). In addition, to prove the potential intravenous administration *in vivo*, we employed the hemolysis assay on red blood cells. No visually red color was found in the supernatant except the color of the suspended PEG-dBSA-RuS_1.7_ NCs that cannot be removed by low-speed centrifugation (Fig. 3b). And the Vis-NIR spectrums result clearly shows there is no characteristic peak of hemoglobin for the samples with different concentrations of PEG-dBSA-RuS_1.7_ NCs, suggesting that the NCs have excellent blood compatibility (Fig. 3b). Furthermore, the treatment with PEG-dBSA-RuS_1.7_ NCs (intravenous (i.v.) injection, up to 14 mg kg^−1^) even for 28 days did not cause death between the PEG-dBSA-RuS_1.7_ NC-treated mice and the control mice. The liver function markers including alanine aminotransferase (ALT), alkaline phosphatase (ALP) and aspartate aminotransferase (AST), and kidney function markers including uric acid (UA) and blood urea nitrogen (BUN), were measured at 1 day, 4 day and 7 day after injection. The presented error bars are based on four mice in each group. The results showed no obvious hepatic and kidney disorder of mice at the given dose (14 mg kg^−1^). Collectively, these results provide evidence of the low *in vitro* and in *vivo* totoxicity of the PEG-dBSA-RuS_1.7_ NCs.

### *In vitro* photothermal ablation of tumor cells

**Figure 4:**
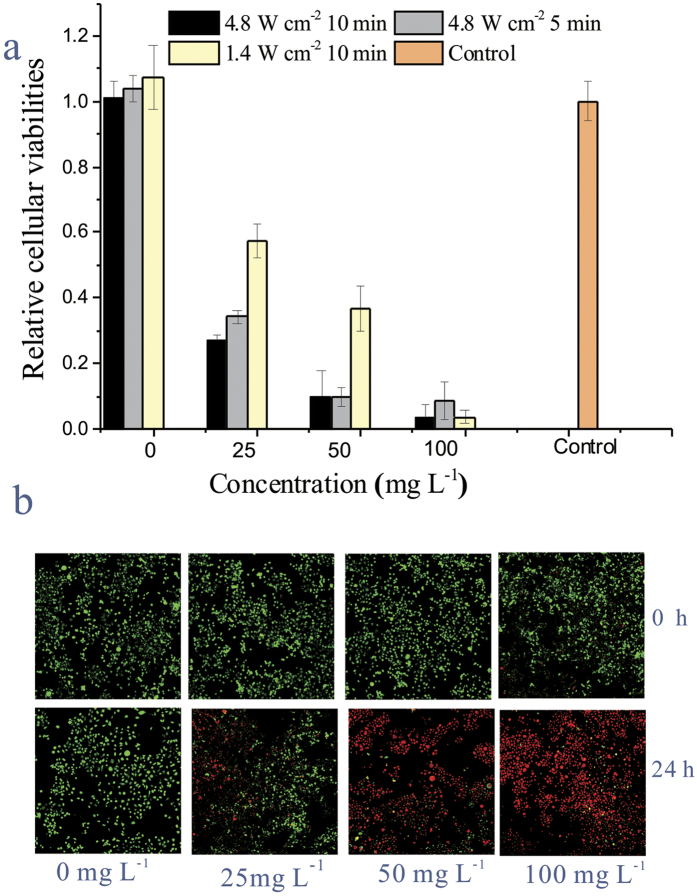
*In vitro* PEG-dBSA-RuS_1.7_ NC-based PTT efficacy of cancer. (**a**) Cell proliferation viabilities of 4T1 cells at 24 h post PTT. (**b**) Live/dead staining of 4T1 cells treated under different concentrations of PEG-dBSA-RuS_1.7_ NCs for 12 h were then observed immediately and 24 hours later after irradiating with an 808-nm laser (1.4 W cm^−2^, 10 min).

Encouraged by low toxicity of PEG-dBSA-RuS_1.7_ NCs, we further tested their PTT efficiency using 4T1 cells as representative cancer cells. Upon 10 min NIR irradiation at power density even up to 4.8 W cm^−2^, the proliferation level determined by the WST1 assay was not decreased for the cells without adding PEG-dBSA-RuS_1.7_ NCs (Fig. 4a). In contrast, for the cancer cells incubated with PEG-dBSA-RuS_1.7_ NCs and subsequently irradiated by NIR laser, the cellular proliferation level gradually decreased with the increase in either laser power density or nanocluster concentration. Typically, when the cells were under a mild laser exposure condition (1.4 W cm^−2^, 10 min) after being incubated with PEG-dBSA-RuS_1.7_ NCs, a small amount of cells died immediately but the massive cell death was observed 24 hours later after irradiation (Fig. 4b). This should be probably caused by the heat-induced apoptosis^36^.

### *In vivo* biodistribution and tumor-targeting efficacy

**Figure 5:**
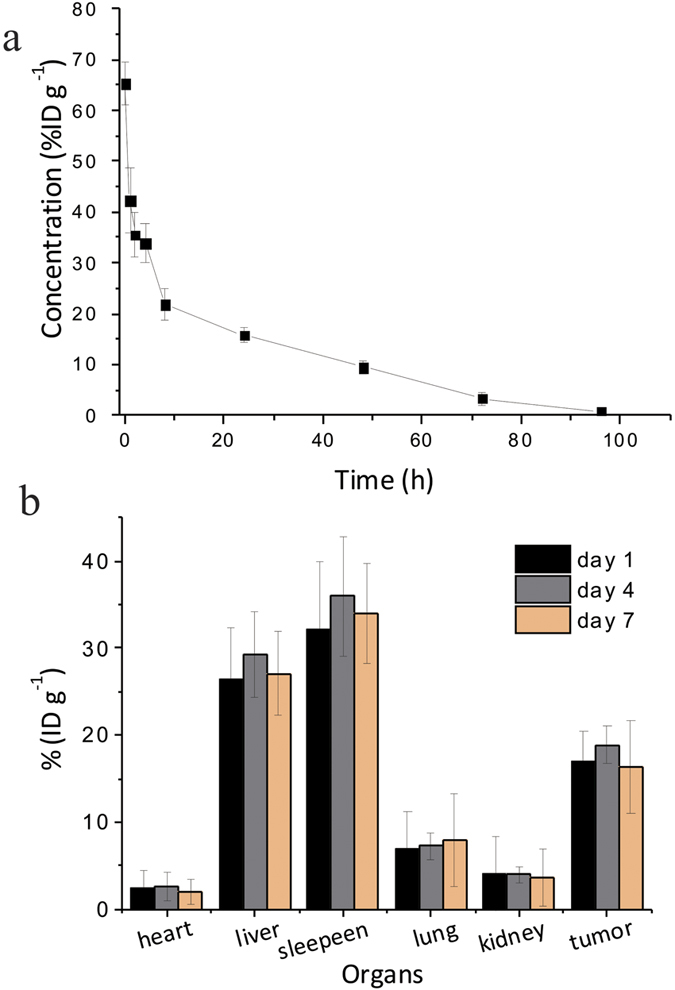
The distribution of PEG-dBSA-RuS_1.7_ NCs in blood and other organs measured by ICP-OES in tumor-bearing mice. Ru concentration levels in blood at different time point (**a**) and in organs at 1, 4 and 7 days after the mice were treated with PEG-dBSA-RuS_1.7_ NCs (14 mg kg^−1^) (**b**).

Before conducting further *in vivo* PTT experiments, it is necessary to confirm the biodistribution of PEG-dBSA-RuS_1.7_ NCs considering that the *in vivo* system is more complicated. Herein, inductively coupled plasma-optical emission spectroscopy (ICP-OES) was employed to determine the Ru amount in the organs and blood that were collected at different intervals from tumor-bearing mice with PEG-dBSA-RuS_1.7_ NC injection. The Ru level in blood was 15.85% ID g^−1^ (percentage of injected dose per gram tissue) at 24 h post injection, which was approximately four times greater than Mo level in the blood of PEGylated MoS_2_ nanosheet-treated mice (3.67% ID g^−1^)^32^. At 72 h post injection, the Ru level in blood was kept at 3.21% ID g^−1^, and Ru was still detectable even at 96 h post injection (0.61% ID g^−1^) (Fig. 5a). These results indicate that the blood circulation time of PEG-dBSA-RuS_1.7_ NCs is very long, which may be attributed to the suitable hydrodynamic size (70 nm) and almost neutral charge. These features effectively enable the PEG-dBSA-RuS_1.7_ NCs to escape from the RES reorganization and avoid renal clearance^28^,^29^. Moreover, the half-life (t_1/2_) of PEG-dBSA-RuS_1.7_ was also calculated to be 2 h which is similar to that of the MoS_2_-GSH nanodots^21^. The long blood circulation time is especially advantageous for the nanoagent accumulation in solid tumor. Indeed, at 4 day post injection the Ru level in tumor was determined to be 18.1% ID g^−1^ (Fig. 5b). It is much higher than that of other TMS nanoagent-treated mice, such as 6.62% for PEGylated MoS_2_ nanosheets^32^; ~2% for Tween-functionalized Bi_2_S_3_ nanorods^24^; and ~12.7% for PEGylated WS_2_ nanoflakes^25^. Within 7 days, the Ru concentration in tumor decreased by 16.3%.

### *In vivo* photothermal ablation of tumor

**Figure 6:**
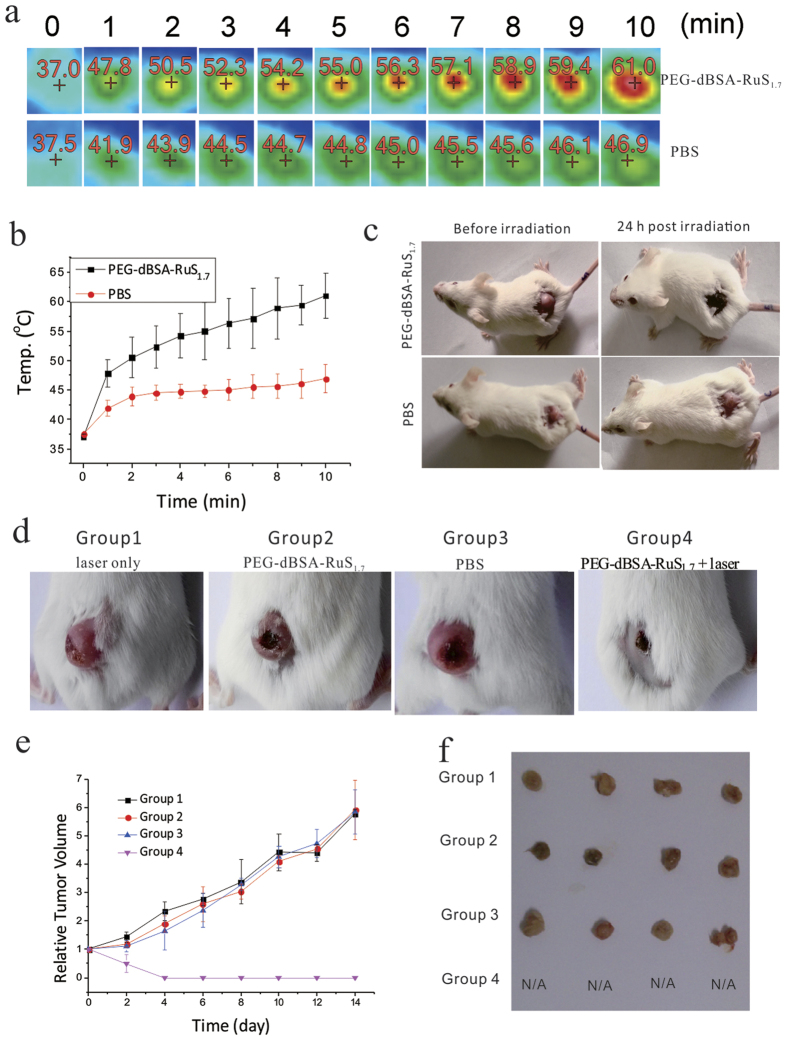
*In vivo* PTT efficacy. (**a**) NIR photothermal images and (**b**) temperature curves of 4T1 tumor-bearing mice at different time intervals with i.v. injection of PEG-dBSA-RuS_1.7_ NCs (dose = 14 mg kg^−1^) and PBS, and then irradiation with the 808 nm laser (1.4 W cm^−2^). (**c**) Photos of the 4T1 tumor-bearing mice with i.v. injection of PEG-dBSA-RuS_1.7_ NCs (dose = 14 mg kg^−1^) and PBS before and 24 h post irradiation. (**d**) Photos of mice taken at 14 day post PTT. Four groups with 4 mice per group: (1) laser only; (2) PEG-dBSA-RuS_1.7_ NCs i.v. injection; (3) PBS i.v. injection; (4) PEG-dBSA-RuS_1.7_ NCs i.v. injection + laser. (**e**) Tumor volume growth curves of different groups of mice as indicated in (**d**); (**f**) The photo showing all tumors collected from all four groups of mice at day 14 post treatment.

Motivated by the high *in vitro* PTT efficacy, the long *in vivo* blood circulation time and high tumor accumulation of PEG-dBSA-RuS_1.7_ NCs, we then carried out the *in vivo* PTT experiments. When the tumor had a width of approximately 5 mm, the female 4T1 tumor-bearing mice were randomized into four groups (n = 4, each group): (1) laser only; (2) PEG-dBSA-RuS_1.7_ NCs i.v. injection; (3) PBS i.v. injection; (4) PEG-dBSA-RuS_1.7_ NCs i.v. injection + laser. Take group (4) as an example, the mice were injected with PEG-dBSA-RuS_1.7_ NC solution (dose = 14 mg kg^−1^), and on the fourth day after injection, the mice were irradiated with an 808 nm NIR laser (1.4 W cm^−2^) for 10 min. The temperature of the tumor area was recorded by an IR thermal camera at 1 min intervals (Fig. 6a,b). Under NIR irradiation, the tumor site temperature of the group (4) rapidly increased from 37 to 61 °C. However, the temperature of the tumor site of the control group (2) only had a small change although the mice were also irradiated by the NIR laser. An obvious crusting was observed in the mice from group (4) at the next day after irradiation while negligible change was detected in those from group (2) (Fig. 6c). Tumors in the control groups (1, 2, and 3) rapidly grew, and no difference in tumor volume was found in these control groups on the 14th day post PTT. By contrast, tumors in the group (4) were no longer observed (Fig. 6d,e,f). These results reveal that PTT based on PEG-dBSA-RuS_1.7_ NCs has exhibited an excellent curative effect and PEG-dBSA-RuS_1.7_ NCs are capable of acting as an ideal photothermal agent for cancer therapy.

## Conclusions

In summary, we have successfully prepared a ruthenium sulfide–based nanomaterial, PEG-dBSA-RuS_1.7_ NCs, which can be applied as a novel photothermal agent with excellent physiological stabilities. The PEG-dBSA-RuS_1.7_ NCs demonstrate good photothermal effects upon NIR laser irradiation, excellent biocompatibility, and no obvious toxicity. More importantly, *in vivo* cancer treatment study further reveals that the as-prepared NCs exhibit quite long blood circulation time and markedly high tumor accumulation compared with many other explored TMS-based photothermal agents, which may benefit from the right hydrodynamic size and almost neutral charge of PEG-dBSA-RuS_1.7_ NCs. As a result, our novel NCs possess the outstanding ability to eradicate the tumor in a mouse model. Our work suggests that as a new class of photothermal agents, ruthenium sulfide-based nanostructures with improved blood residence and tumor uptake may facilitate the *in vivo* applications of TMS-based PTT.

## Methods

### Ethics Statement

All animal experiments were performed with the approval by Hainan Medical College, and in compliance with the principles stated in the Guide for the Care and Use of Laboratory Animals, National Research Council.

### Material Preparation

Calcein-AM and propidium iodide (PI) were provided by Invitrogen, mPEG-NH_2_ (MW = 5 K) was purchased from Seebio Co. Ltd., Shanghai, China, and other chemicals were purchased from Sigma-Aldrich. The dBSA were synthesized according to our previous report^37^. All the chemicals were used as received without further purification.

### Preparation of OA-coated RuS_1.7_ NDs

The OA-RuS_1.7_ NDs were prepared by a solvothermal method. For a typical synthesis, Ru(DDTC)_3_ was firstly prepared according to the previous literature^38^ by mixing 100 mmol of RuCl_3_ and 300 mmol of diethyl dithiocarbamate (Na(DDTC)) in 500 mL of distilled water. After constant stirring for 1 h, the solution was kept stationary under ambient condition for another 3 h. The resulting precipitate was filtered, washed with distilled water and dried in air at 50 °C for 24 h to obtain Ru(DDTC)_3_. Then, 272 mg of Ru(DDTC)_3_ was dissolved with 10 mL of ethyl alcohol. After stirring for 30 min, 20 mL of OA was added and kept stirring for another 15 min. The Ru(DDTC)_3_ colloidal solution was then transferred to a 50 mL Teflon-lined autoclave, sealed, and heated at 200 °C for 12 h. The mixture was then cooled to room temperature. The products were collected by centrifugation at 20000 g and washed with ethyl alcohol several times to remove the remnant. The final products were dissolved with 40 mL of chloroform.

### Sequential dBSA and PEG Modifications of RuS_1.7_ NDs

To transfer RuS_1.7_ NDs from an organic phase to an aqueous phase, sequential dBSA and PEG modifications were conducted according to our previous report^37^. In a typical procedure, 10 mg of OA-RuS_1.7_ NDs was dispersed in 9 mL of chloroform. Then 100 mg of dBSA dissolved with 1 mL of dimethyl sulfoxide (DMSO) was added to the mixture. After stirring overnight at room temperature, dBSA coated RuS_1.7_ NCs were collected by centrifuging the mixture at 50000 g and washing with distilled water. The purified dBSA-RuS_1.7_ NCs were dispersed with 5 mL of DMSO. For PEG conjugation, 50 mg of mPEG-NH_2_ (MW = 5 K) and 20 mg of 1-(3-Dimethylaminopropyl)-3-ethylcarbodiimide hydrochloride (EDAC) were added to dBSA-RuS_1.7_ NCs under vigorous stirring overnight at room temperature. The as-synthesized PEG-dBSA-RuS_1.7_ NCs were intercepted using a 100 kDa ultra-filter and washed with distilled water. The final purified PEG-dBSA-RuS_1.7_ NCs were redispersed with distilled water and filtered by a membrane with 0.22 μm pore size.

### Characterization of RuS_1.7_ NDs and PEG-dBSA-RuS_1.7_ NCs

The morphology and size of the as-prepared nanoparticles were characterized by the transmission electron microscope (TEM, JEM-2100, JOEL). Vis-NIR spectrum was recorded by a UV spectrophotometer (UV2600, Shimadzu, Japan). X-ray diffraction (XRD) spectrum of the dry powder was measured using an ARL-X’TRA x-ray diffractometer. X-ray photoelectron spectra were obtained on a PHI 5000 VersaProbe XPS spectrometer. X-ray fluorescence spectra were recorded on an ARL-9800 x-ray fluorescence spectrometer.

### Cytotoxicity and *in vitro* PTT Effect Study

4T1 and L929 cell lines were cultured in 1640 cell medium contained 10% fetal bovine serum (FBS) at 37 °C and a 5% CO_2_ atmosphere. Cells were seeded into 96-well plates at a density of 10^4^ cells per well, and were incubated with different concentrations of PEG-dBSA-RuS_1.7_ NCs for 24 or 48 h. Relative cellular viabilities were measured by the standard WST-1 assay. For *in vitro* PTT, 4T1 cells were incubated with various concentrations of PEG-dBSA-RuS_1.7_ NCs for 12 h and then irradiated by an 808 nm laser at a series of power densities with different exposure times. At 24 h post laser exposure, the cells were stained with calcein AM and PI for 15 min, then imaged by a confocal fluorescence microscope (Olympus). For detecting cell proliferating viability, a standard WST1 assay was conducted at 24 h post laser exposure.

### Hemolysis Assay of PEG-dBSA-RuS_1.7_ NCs

Red blood cells were obtained by removing the serum from BALB/c female mice blood after being washed with 0.9% saline, and centrifuged five times. After that, blood cells were diluted to 1:10 with PBS solution. Then, 0.2 mL of diluted cells suspension was mixed with 0.8 mL of PBS (as a negative control), 0.8 mL of deionized water (as a positive control), and 0.8 mL of product suspensions. The samples were shaken and kept steady for 2 h. Finally, the mixtures were centrifuged at 1000 g for 5 minutes and the absorbance of the upper supernatants was measured by UV-vis spectroscopy.

### Animals and preparation of tumor-bearing mice

BALB/c female mice with body weights of about 20 g were obtained from Tianqin Biotechnology Co., Ltd, Changsha, China, and housed under protocols approved by Hainan Medical College. Tumor-bearing mice were prepared by inoculating 10^6^ 4T1 cells in the backside of each mouse.

### Inductively coupled plasma-optical emission spectroscopy (ICP-OES) for Ru element quantification

To quantify the tissue-distribution of PEG-dBSA-RuS_1.7_ NCs, the NCs (14 mg kg^−1^) were i.v. injected to the tail vein of tumor-bearing BALB/c mice. The mice (n = 4) were euthanized at different time points (1, 4 and 7 days). Then tissue samples were stored at −20 °C before analysis. For ICP-OES experiment, each sample was added with 0.4 mL of aqua regia and digested for 48 h at 60 °C. The final solutions were diluted to 1 mL and filtered. Ru contents of all samples were measured by ICP-OES.

### Assessing *in vivo* PTT efficacy of PEG-dBSA-RuS_1.7_ NCs

When the tumor size reached approximately 5 mm in width, the mice were divided into four groups: (a) blank; (b) laser only; (c) PEG-dBSA-RuS_1.7_ NCs i.v. injection; and (d) PEG-dBSA-RuS_1.7_ NCs (i.v. injection) + laser. The 4T1 tumor-bearing mice were i.v. injected with PEG-dBSA-RuS_1.7_ NCs (dosage = 14 mg kg^−1^) and exposed to an 808 nm laser with a 1 W cm^−2^ of power density for 10 min. Temperature change under laser irradiation in the tumor site was recorded by a NIR camera. Photos were captured at every 1 minute interval. Tumorswere measured in the following days, and tumor sizes were calculated as follows^24^


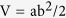


where V (mm^3^) is the tumor volume, and a (mm) and b (mm) are the tumor length and width, respectively.

## Additional Information

**How to cite this article:** Lu, Z. *et al*. Long Blood Residence and Large Tumor Uptake of Ruthenium Sulfide Nanoclusters for Highly Efficient Cancer Photothermal Therapy. *Sci. Rep.* **7**, 41571; doi: 10.1038/srep41571 (2017).

**Publisher's note:** Springer Nature remains neutral with regard to jurisdictional claims in published maps and institutional affiliations.

## Supplementary Material

Supplementary Information
