# Supplementary material for: Long Blood Residence and Large Tumor Uptake of Ruthenium Sulfide Nanoclusters for Highly Efficient Cancer Photothermal Therapy
Source: Sci Rep. 2017 Jan 31;7:41571. doi: 10.1038/srep41571 (PMC5282482; doi:10.1038/srep41571)
Supplement: Supplementary Information [file srep41571-s1.doc]

Supplementary Information

Long Blood Residence and Large Tumor Uptake of Ruthenium Sulfide Nanoclusters for Highly Efficient Cancer Photothermal Therapy

**Zhuoxuan Lu^1^, *, Feng-ying Huang^1^, *, Rong Cao^2^, Liming Zhang^1^, Guang-hong Tan^1^ , Nongyue He^3^, Jie Huang^4^, Guizhen Wang^5^, and Zhijun Zhang^4^**

^1^ Key Laboratory of Tropical Disease and Translational Medicine of the Ministry of Education, Hainan Medical College, Haikou 571101, China

^2^ Department of Chemical Engineering, Monash University, Wellington Rd., Clayton, Vic 3800, Australia

^3^ Hunan Key Laboratory of Green Chemistry and Application of Biological Nanotechnology, Hunan University of Technology, Zhuzhou 412008, China

^4^ Key Laboratory of Nano-Bio Interface, Division of Nanobiomedicine, Suzhou Institute of Nano-tech and Nano-bionics, Chinese Academy of Sciences, Suzhou 215123, China

^5^ College of Materials and Chemical Engineering, Hainan University, Haikou 570228, China

*These authors contributed equally to this work.

Correspondence and requests for materials should be addressed to Z. L, (email: [lmzhang1980@163.com](mailto:lmzhang1980@163.com)) or T. G. (email: [tanhoho@163.com](mailto:tanhoho@163.com)) or H. N. (email: nyhe1958@163.com)


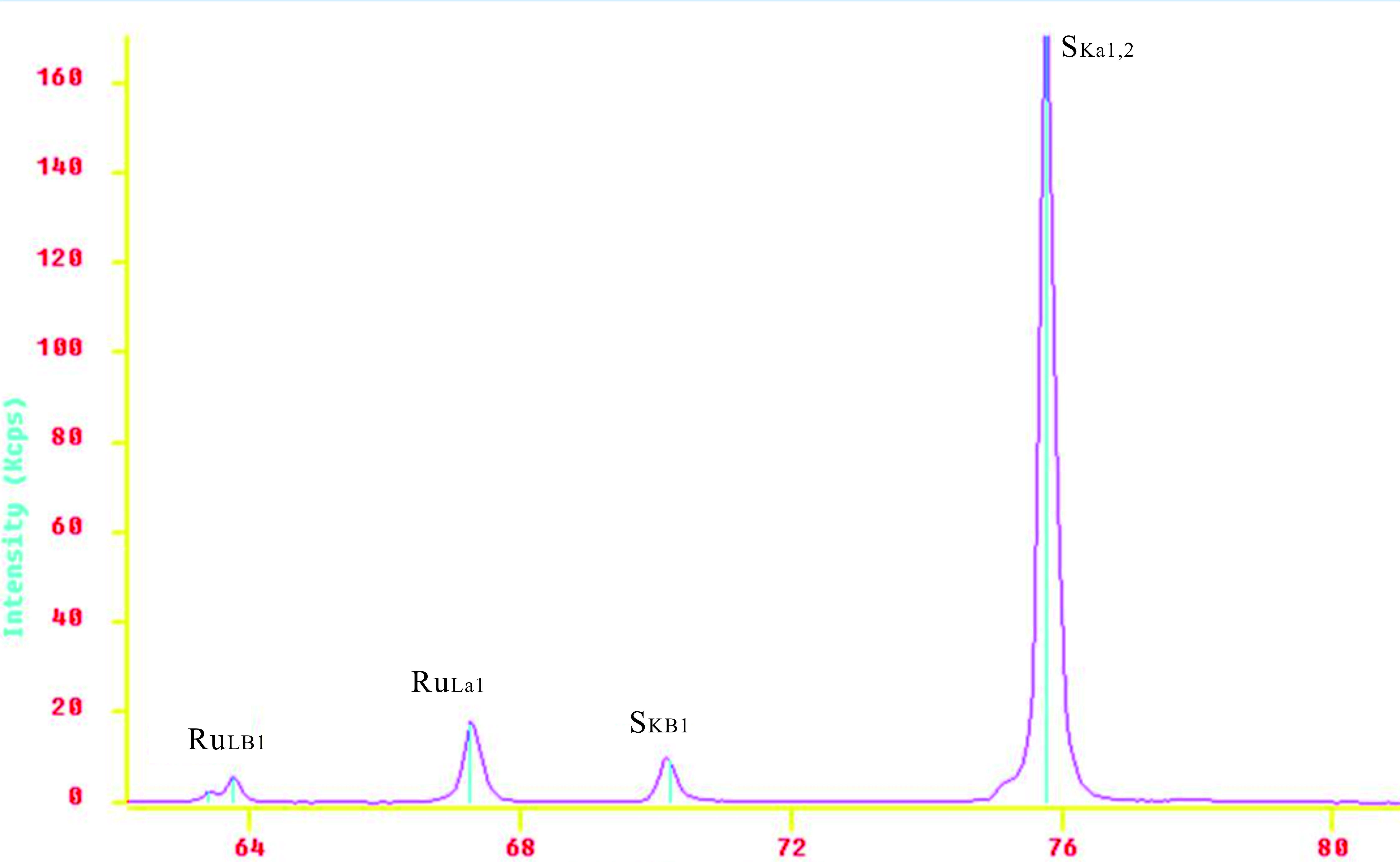


Figure S1. XRF spectrum of RuS_1.7_ NDs.

Figure S2. XRD spectrum of RuS_1.7_ NDs.

Figure S3. XPS spectra of RuS_1.7_ NDs: Ru 3d spectrum (a) and S 2p spectrum (b).

Figure S4. FTIR spectra of OA and PEG-dBSA coated RuS_1.7_ NDs.


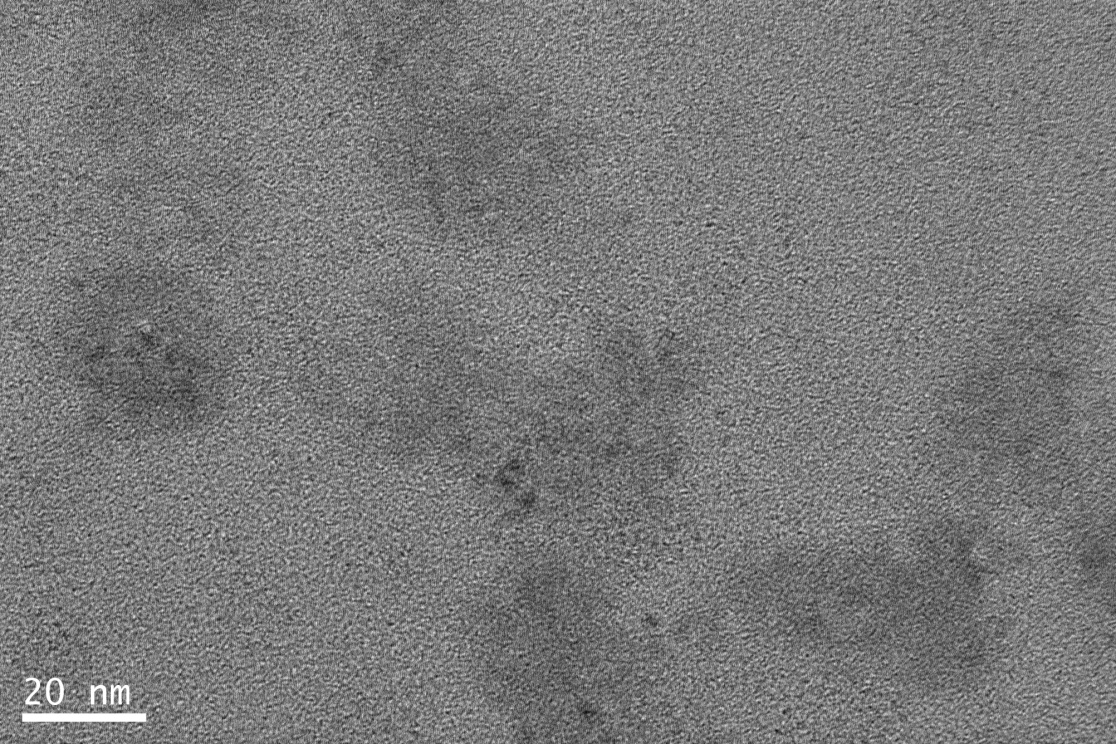


Figure S5. TEM image of PEG-dBSA-RuS_1.7_ NCs.


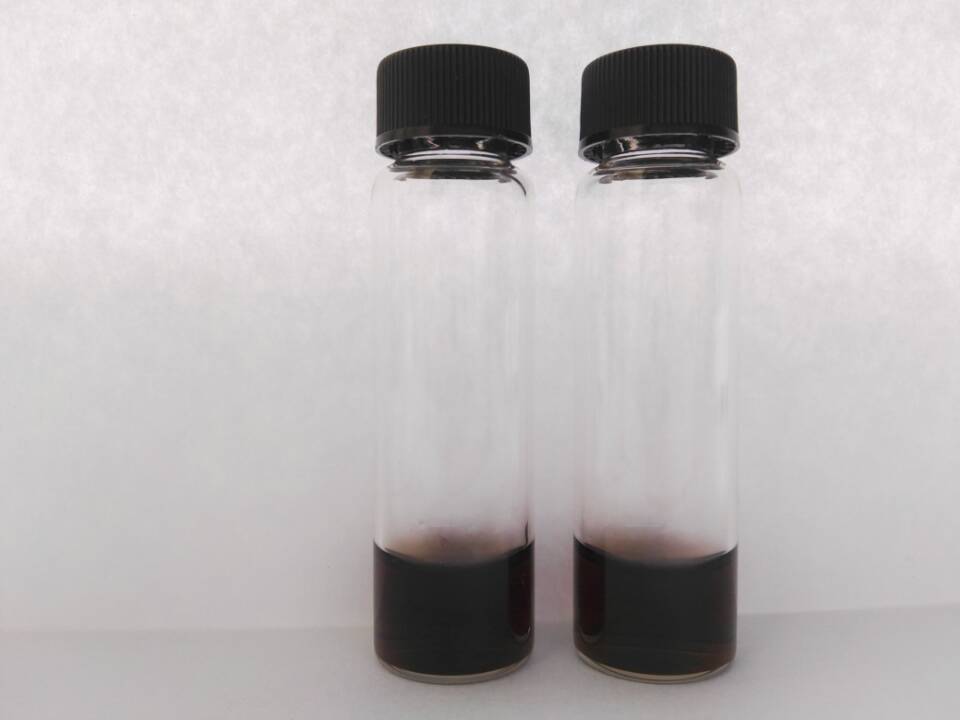


a

b

Figure S6. The stability of PEG-dBSA-RuS_1.7_ NCs in water (a) and PBS (b).

Figure S7. Zeta potential of PEG-dBSA-RuS_1.7_ NCs.

**Table S1.** Blood biochemistry analysis of the mice treated with PEG-dBSA-RuS_1.7_ NCs. The results show mean and standard deviation of alanine aminotransferase (ALT), alkaline phosphatase (ALP) aspartate aminotransferase (AST), uric acid (UA) and blood urea nitrogen (BUN).

|  | ALT  (U/L) | ALP  (U/L) | AST  (U/L) | UA  (µmol/L) | BUN  (mmol/L) |
| --- | --- | --- | --- | --- | --- |
| Day 1 | | | | | |
| PBS | 36.5±8.95 | 151±20.5 | 135±17.7 | 128±10.6 | 9.02±1.78 |
| PEG-dBSA-RuS_1.7_ | 42.5±7.85 | 148±17.9 | 132±30.0 | 142±17.7 | 10.8±0.25 |
| Day 4 | | | | | |
| PBS | 38.5±10.7 | 152±13.5 | 129±21.5 | 121±21.3 | 8.98±0.87 |
| PEG-dBSA-RuS_1.7_ | 35.2±11.6 | 146±34.8 | 136±23.7 | 137±26.7 | 10.3±1.52 |
| Day 7 | | | | | |
| PBS | 33.7±7.8 | 155±36.3 | 141±27.5 | 124±23.9 | 10.1±0.61 |
| PEG-dBSA-RuS_1.7_ | 39.7±6.68 | 149±32.1 | 134±21.6 | 141±30.7 | 9.39±1.08 |
|  |  |  |  |  |  |
